# Supplementary material for: Fish are poor sentinels for surveillance of riverine antimicrobial resistance
Source: One Health. 2025 Apr 1;20:101026. doi: 10.1016/j.onehlt.2025.101026 (PMC11999348; doi:10.1016/j.onehlt.2025.101026)
Supplement: Supplementary file 1 — Supplementary Figures S1-S6 [file mmc1.docx]

## **Supplementary information for the manuscript:**

**Fish are poor sentinels for surveillance**

**of riverine antimicrobial resistance**

Faina Tskhay^1^, Christoph Köbsch^1^, Alan X. Elena^1^, Johan Bengtsson-Palme^2,3,4^, Thomas U. Berendonk^1^, Uli Klümper^1,#^

^1^ Institute of Hydrobiology, Technische Universität Dresden, 01217 Dresden, Zellescher Weg 40, Germany

^2^ Division of Systems and Synthetic Biology, Department of Life Sciences, SciLifeLab, Chalmers University of Technology, SE-412 96 Gothenburg, Sweden
^3^ Department of Infectious Diseases, Institute of Biomedicine, The Sahlgrenska Academy, University of Gothenburg, Guldhedsgatan 10A, SE-413 46 Gothenburg, Sweden
^4^ Centre for Antibiotic Resistance Research (CARe) in Gothenburg, Sweden

^#^ corresponding author

### Corresponding author

Dr. Uli Klümper (ORCID: 0000-0002-4169-6548)

Technische Universität Dresden, Institute of Hydrobiology,

01062 Dresden,

Zellescher Weg 40,

Germany

E-mail: Uli.Kluemper@TU-Dresden.de

Phone: +49 351 463 43273

**
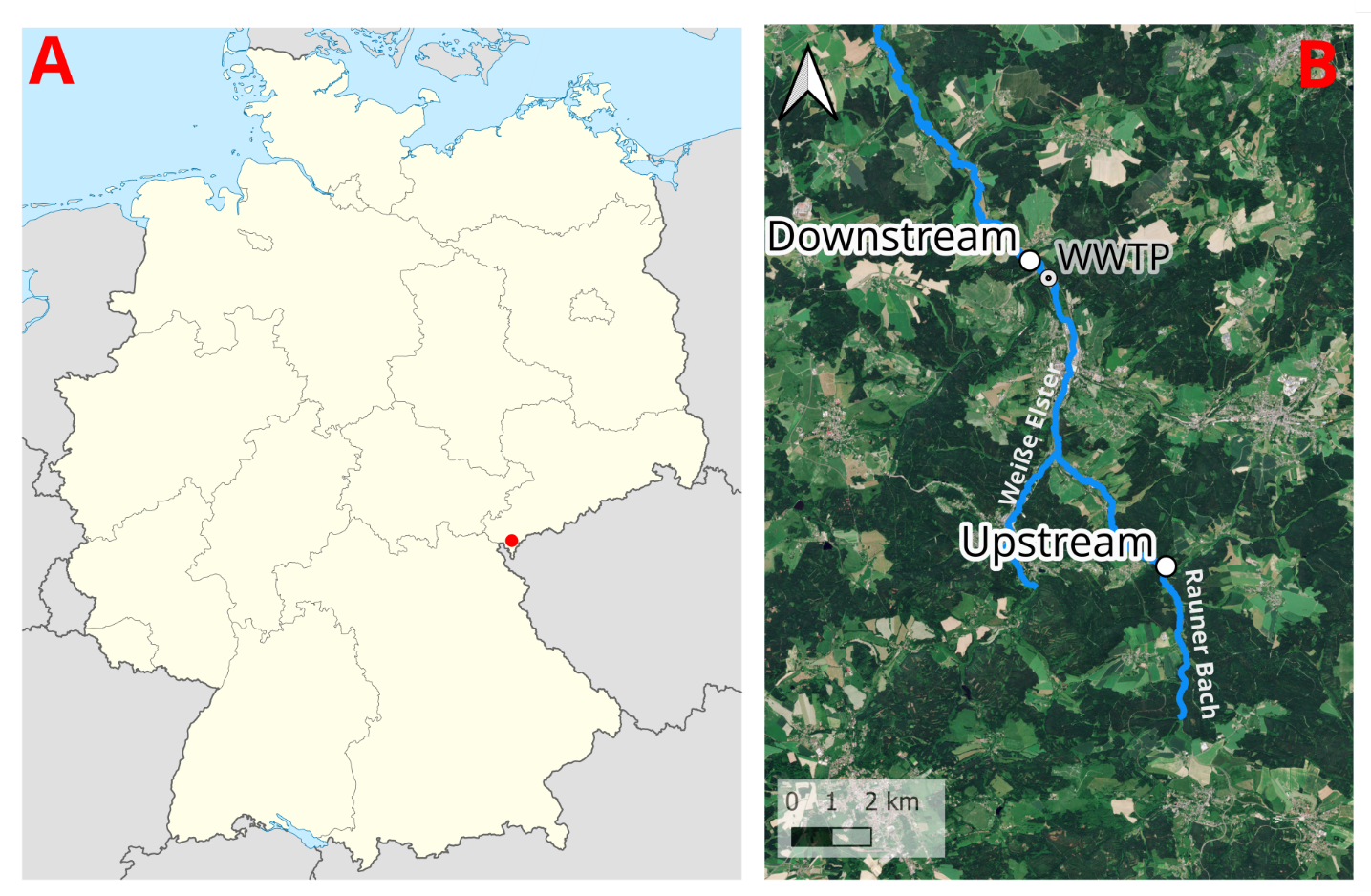
**

**Figure S1: Map of the sampling locations. A) Map of Germany with the location of the study area, the Weiße Elster – Rauner Bach tributary in Vogtland, Saxony marked with a red dot. The map image was obtained under the terms of the GNU Free Documentation License, Version 1.2 from [1]. B) Satellite image displaying the location of the wastewater treatment plant (WWTP) and the sampling locations upstream (approximately 7.6 km) and downstream (approximately 0.8 km) of the WWTP. The map was generated in ArcGIS Pro (Esri, Redlands, CA, USA) software using open-source location data. The satellite image was generated from the Sen2Europe Map (© European Union, contains Copernicus Sentinel-2 data [2025], processed by the Federal Agency for Cartography and Geodesy (BKG)). River open-source data was obtained from Gewässernetz - LUIS (Sächsisches Landesamt für Umwelt, Landwirtschaft und Geologie) [2] and integrated into the map.**

**
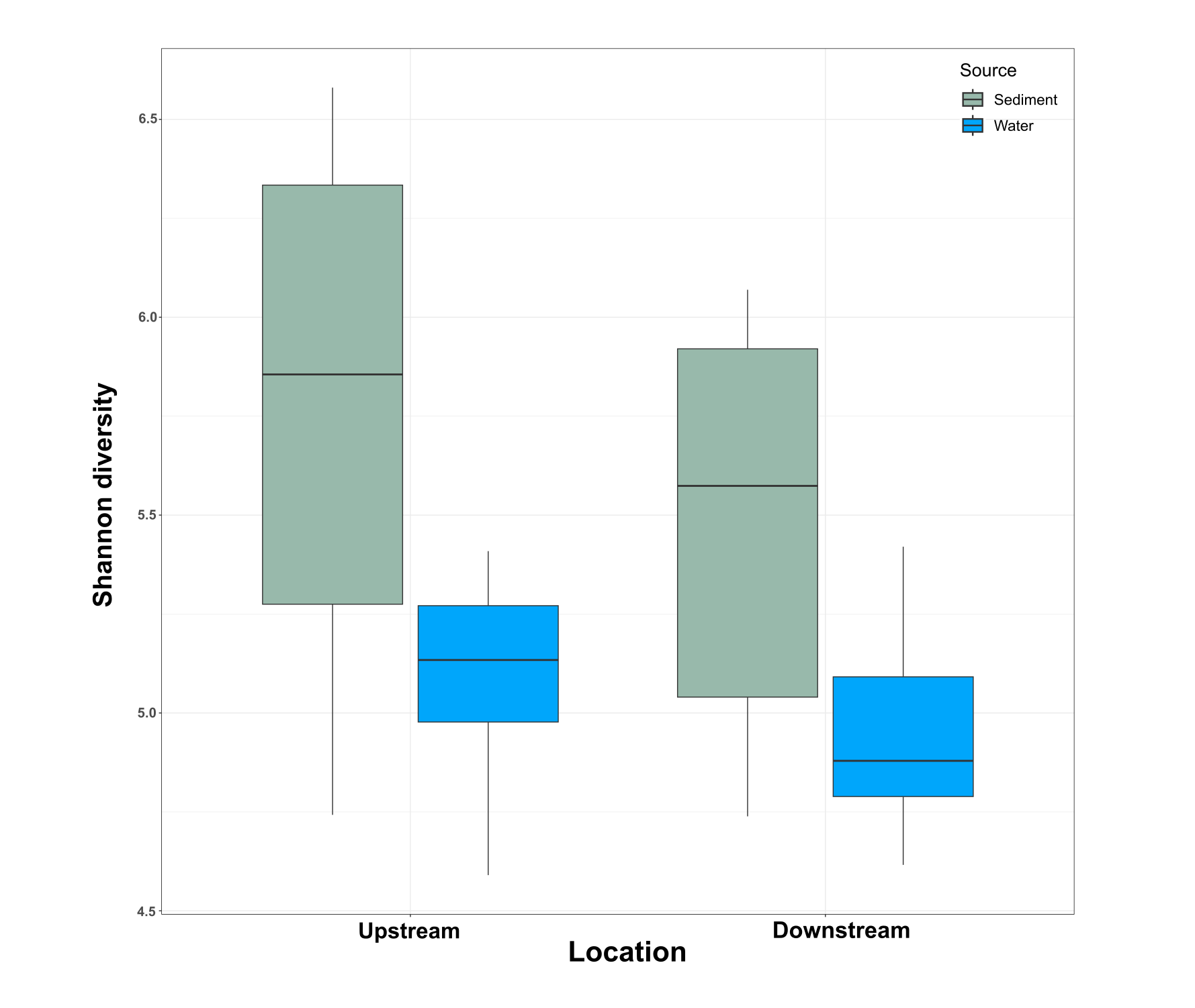
**

**Figure S2. Shannon diversity index for water (A) and sediment (B) samples collected up- and downstream of the WWTP. The boxplots show the distribution of the Shannon index (H) at each location relative to the WWTP. The horizontal line indicates the median diversity value for each location.**


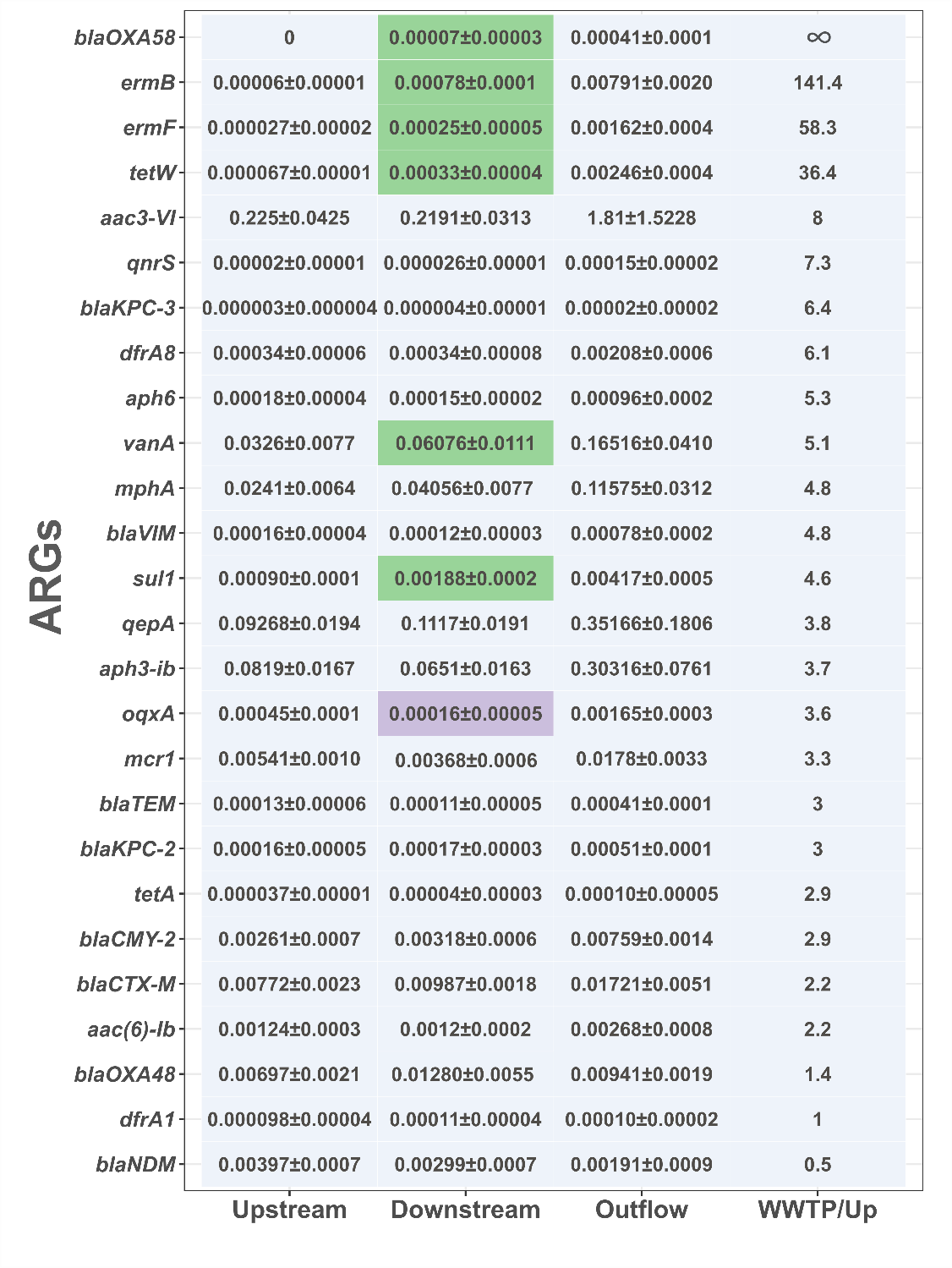


**Figure S3. Relative abundance of the ARGs in water samples. The numbers represent the average relative abundance of six biological replicates, with standard deviation. The WWTP/Upstream ratio was calculated by dividing the average relative abundance of each gene upstream of the WWTP by the relative abundance of the WWTP effluent. ARGs are presented in descending order of their P >WWTP/Upstream ratio. Ratios, where the ARG was not detected upstream but was detected in the WWTP effluent, are given as ∞. A two-tailed t-test with Bonferroni correction for multiple testing was performed to test the statistical significance of the difference in relative abundance of ARGs up- and downstream of the WWTP. Relative abundance highlighted in green indicates those ARGs with a statistically significant increase of the relative abundance downstream of the WWTP (P<0.05). Relative abundance highlighted in purple shows the ARGs with a statistically significant decrease in the relative abundance of the ARGs (P<0.05). This supplementary figure provides a detailed representation of the data presented in Fig. 6, particularly regarding the average relative abundance of ARGs per one copy of the 16S rRNA gene, along with standard deviations.**

**
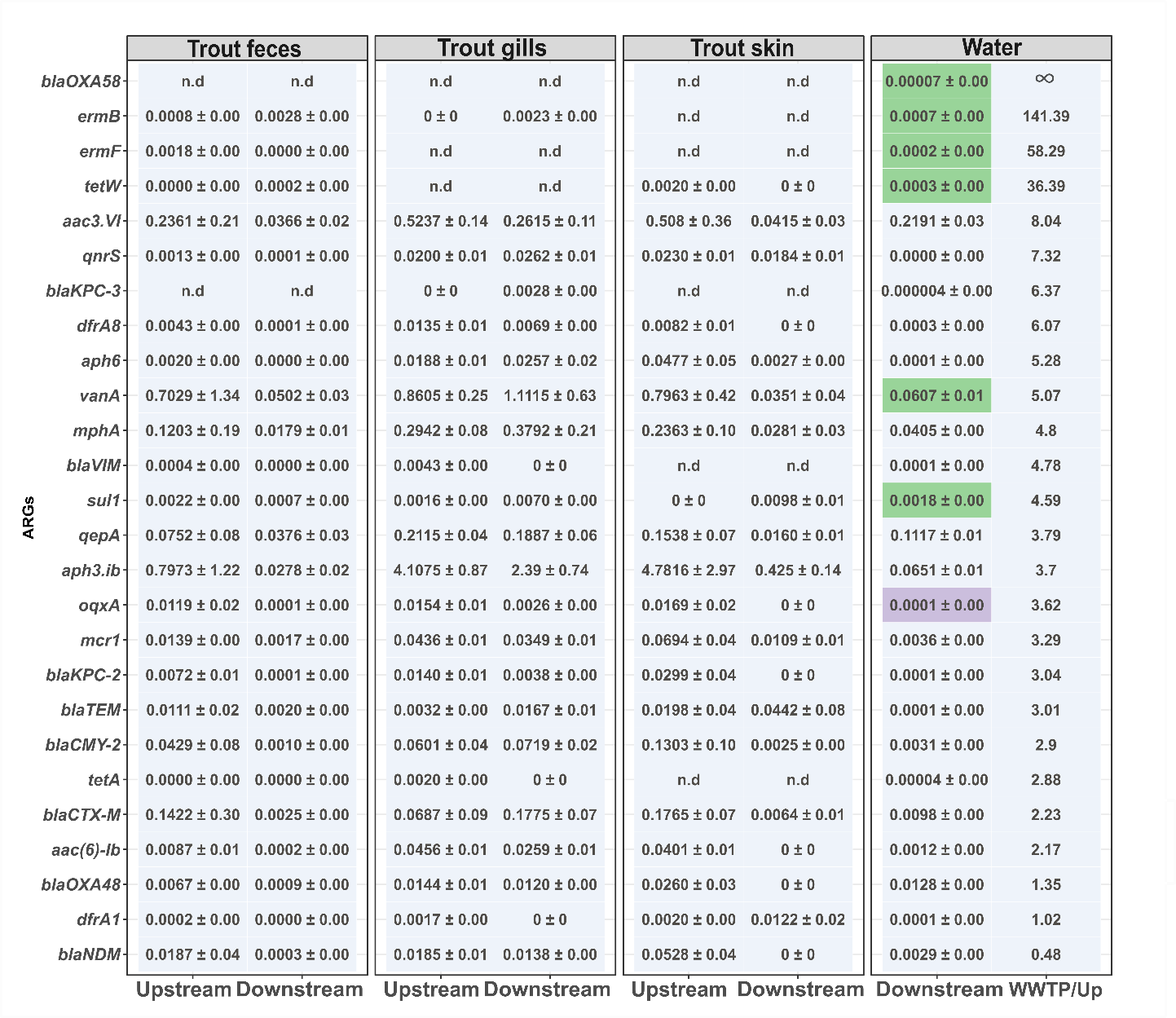
Figure S4: Relative abundance of the ARGs in trout samples. The numbers represent the average relative abundance of the biological replicates, with standard deviation. A two-tailed t-test with Bonferroni correction for multiple testing was performed to test the statistical significance of the difference in relative abundance of ARGs up- and downstream of the WWTP. Relative abundance highlighted in green indicates the ARGs with a statistically significant increase of the relative abundance downstream of the WWTP (P<0.05). Relative abundance highlighted in purple is the ARGs with a statistically significant decrease in the relative abundance of the ARGs (P<0.05). The column with the relative abundance of the ARGs observed in water downstream of the WWTP indicates the resistance genes, whose increase in relative abundance was impacted by the effluent. Ratios, where the ARG was not detected in water samples upstream of the WWTP but was detected in the WWTP effluent, are given as ∞. This supplementary figure provides a detailed representation of the data presented in Fig. 7, particularly regarding the average relative abundance of ARGs per one copy of the 16S rRNA gene, along with standard deviations.**


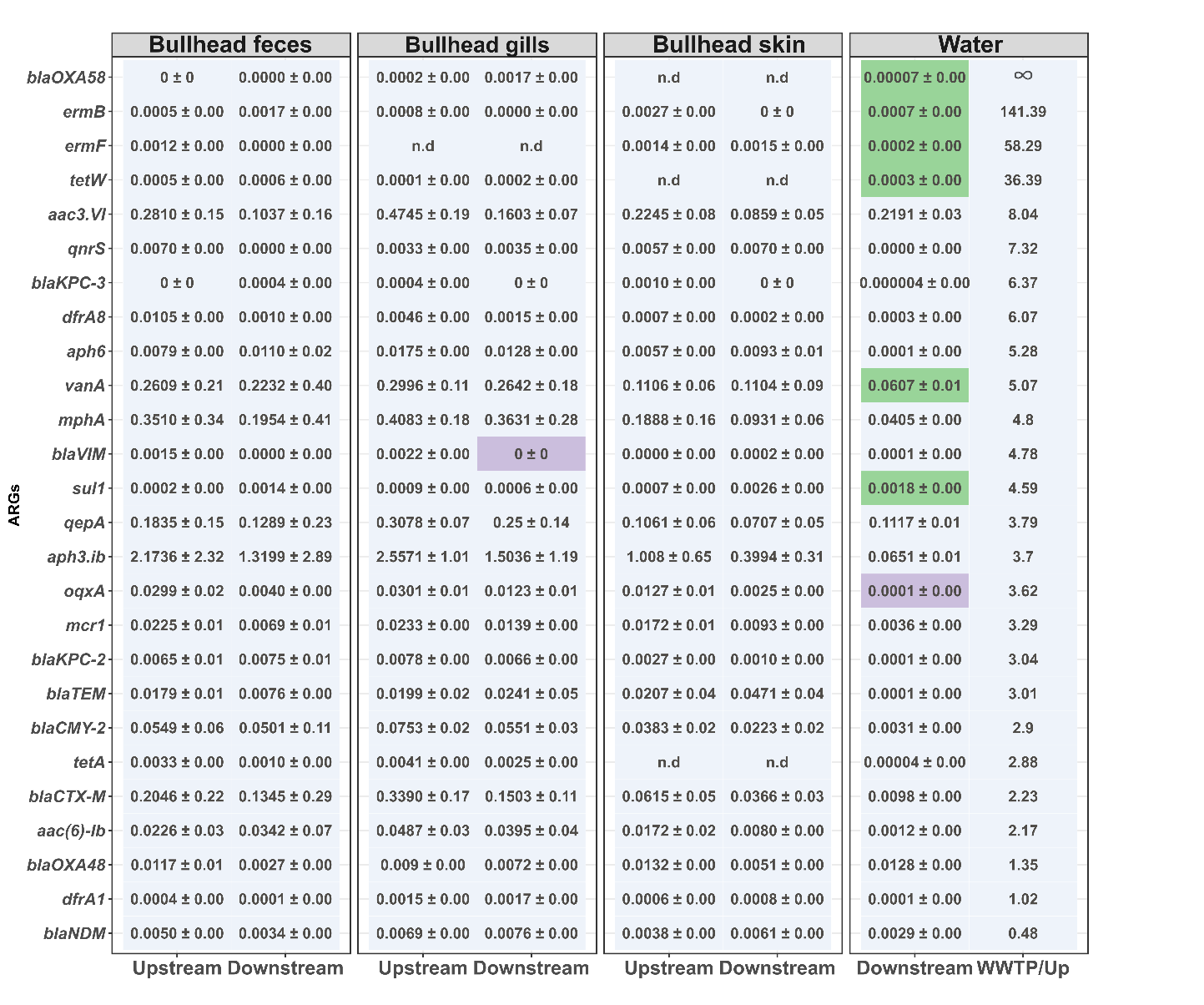


**Figure S5. Relative abundance of the ARGs in bullhead samples. The numbers represent the average relative abundance of the biological replicates, with standard deviation. A two-tailed t-test with Bonferroni correction for multiple testing was performed to test the statistical significance of the difference in relative abundance of ARGs up- and downstream of the WWTP. Relative abundance highlighted in green indicates the ARGs with a statistically significant increase of the relative abundance downstream of the WWTP (P<0.05). Relative abundance highlighted in purple is the ARGs with a statistically significant decrease in the relative abundance of the ARGs (P<0.05). The column with the relative abundance of the ARGs observed in water downstream of the WWTP indicates the resistance genes, whose increase in relative abundance was impacted by the effluent. Ratios, where the ARG was not detected in water samples upstream of the WWTP but was detected in the WWTP effluent, are given as ∞.**


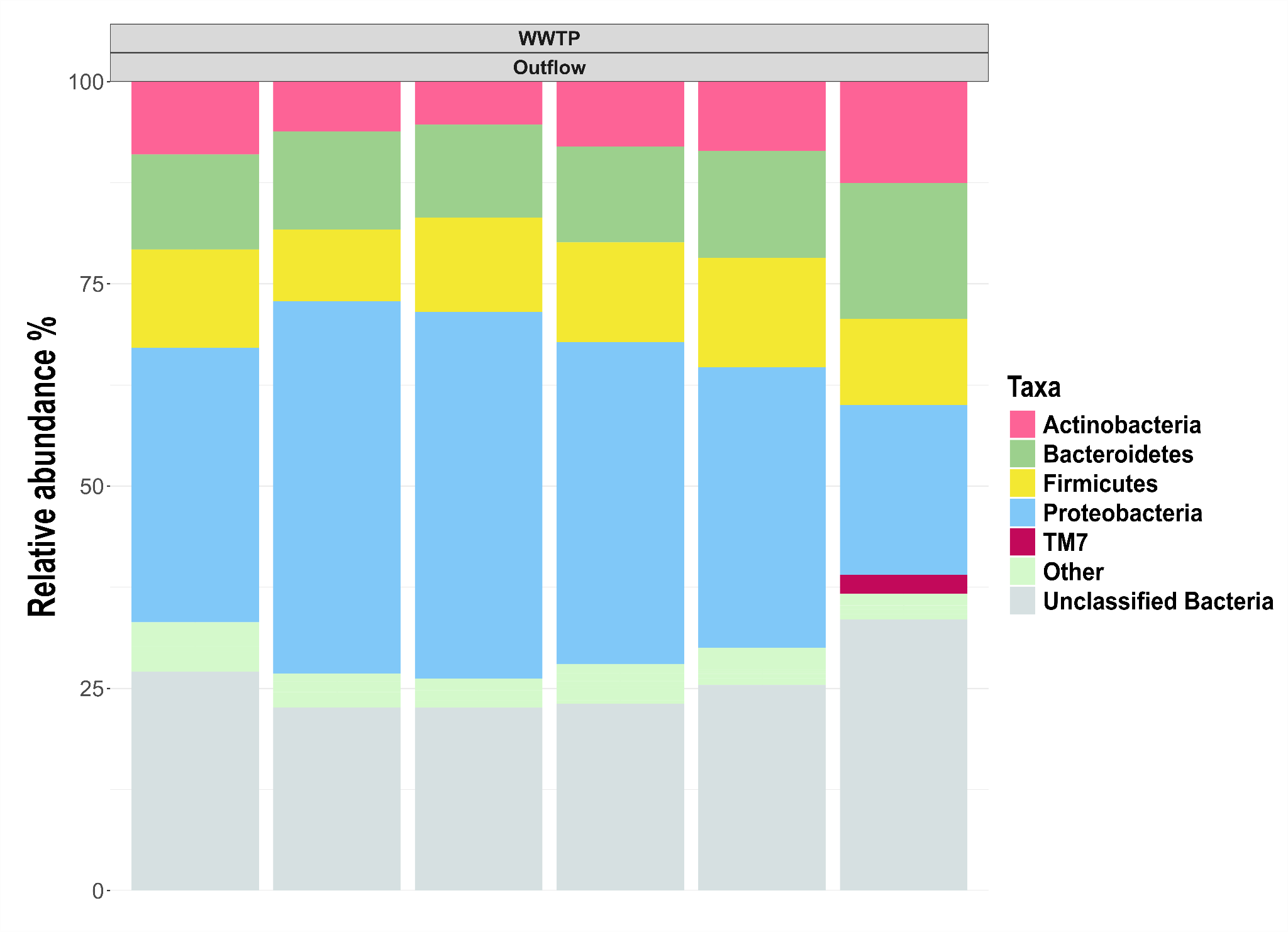


**Figure S6: Relative abundance of the phyla dominated in bacterial community composition of the wastewater effluent across six replicate samples. Dominant phyla are defined as those with an average relative abundance of more than 2%. The remaining phyla are grouped as “Other”.**

## References for the supplementary information

[1] NordNordWest, Deutsch: Positionskarte von Deutschland (de-Wikipedia-Artikel), 2008. https://commons.wikimedia.org/wiki/File:Germany_location_map.svg (accessed March 3, 2025).

[2] Sächsisches Landesamt für Umwelt, Landwirtschaft und Geologie, Gewässernetz (Fließgewässer und Standgewässer) - LUIS - Landwirtschaft- und Umweltinformationssystem für Geodaten, (2025). https://luis.sachsen.de/wasser/gewaessernetz.html (accessed March 2, 2025).
